# Urinary Triclosan is Associated with Elevated Body Mass Index in NHANES

**DOI:** 10.1371/journal.pone.0080057

**Published:** 2013-11-21

**Authors:** Joanna Lankester, Chirag Patel, Mark R. Cullen, Catherine Ley, Julie Parsonnet

**Affiliations:** 1 Department of Electrical Engineering, Stanford University, Stanford, California, United States of America; 2 Department of Medicine, Stanford University School of Medicine, Stanford, California, United States of America; 3 Department of Pediatrics, Stanford University School of Medicine, Stanford, California, United States of America; 4 Department of Health Research and Policy, Stanford University School of Medicine, Stanford, California, United States of America; Brigham & Women's Hospital, and Harvard Medical School, United States of America

## Abstract

**Background:**

Triclosan—a ubiquitous chemical in toothpastes, soaps, and household cleaning supplies—has the potential to alter both gut microbiota and endocrine function and thereby affect body weight.

**Methods:**

We investigated the relationship between triclosan and body mass index (BMI) using National Health and Nutrition Examination Surveys (NHANES) from 2003–2008. BMI and spot urinary triclosan levels were obtained from adults. Using two different exposure measures—either presence vs. absence or quartiles of triclosan—we assessed the association between triclosan and BMI. We also screened all NHANES serum and urine biomarkers to identify correlated factors that might confound observed associations.

**Results:**

Compared with undetectable triclosan, a detectable level was associated with a 0.9-point increase in BMI (*p*<0.001). In analysis by quartile, compared to the lowest quartile, the 2nd, 3rd and 4th quartiles of urinary triclosan were associated with BMI increases of 1.5 (*p*<0.001), 1.0 (*p* = 0.002), and 0.3 (*p* = 0.33) respectively. The one strong correlate of triclosan identified in NHANES was its metabolite, 2,4-dichlorophenol (ρ = 0.4); its association with BMI, however, was weaker than that of triclosan. No other likely confounder was identified.

**Conclusions:**

Triclosan exposure is associated with increased BMI. Stronger effect at moderate than high levels suggests a complex mechanism of action.

## Introduction

Triclosan, also known as 5-chloro-2-(2,4-dichlorophenoxy)phenol, is a broad-spectrum phenolic biocide with activity against bacteria and fungi. It was first licensed for use in the 1960s and has rapidly become a ubiquitous human exposure. This chemical and its related congener triclocarban are impregnated in a wealth of household products ranging from soaps to children's toys. Most triclosan exposure is due to the use of triclosan-containing personal care products [1]. Although toothpaste with triclosan reduces plaque and gingivitis [2], no other benefits to human health have yet been established to support its widespread use in other personal care and household cleaning products. Yet, despite this lack of proven efficacy, triclosan is so widely employed that 75% of human urine samples from both U.S. [3] and Belgian population surveys [4] contained this biocide. In the environment, triclosan has been found in 50% of surface water samples [5] where its potential adverse effects on the aqueous flora have been of concern [6], [7]. In experimental animal models, but not in human studies, triclosan has been reported to be an endocrine disruptor [8] and to affect thyroid [9], [10], estrogen [10], [11], and testosterone [12] levels. For these reasons, triclosan has come under increasing scrutiny by U.S. and European regulatory agencies.

Triclosan is presumed to reduce plaque and gingivitis by altering the composition of the oral flora. At low concentrations, triclosan inhibits fatty acid synthesis by binding to enoyl-acyl carrier protein reductase, preventing normal formation of bacterial cell membranes. At higher, biocidal levels, triclosan destabilizes cell membranes, leading to rapid cell death [13]. Through these mechanisms, it is biocidal against Gram-positive, Gram-negative, aerobic and anaerobic bacteria, although both natural (Pseudomonas, Bacillus) and acquired resistance (*e.g.*, MRSA, *E. coli*, Acinetobacter with Fabl mutations) have been observed [14]. Its detection in blood and urine indicates that it is absorbed and has the potential to have even broader effects on the human microbiota than simply in the mouth and on the skin.

Accumulating data suggest a role for the colonizing microbiota in determining body mass [15], [16], [17], [18]. The precise mechanism by which alteration of the gut flora affects weight remains unknown. Among the prevalent hypotheses are: increased energy extracted from ingested food, modified mechanism of fat storage, inflammation, and alteration in appetite [19], [20], [21], [22]. Although human studies have not eliminated the confounding effects of diet, controlled experiments in animals demonstrate profound effects of gut flora on weight [23], [24], [25]. Because the rapid rise in obesity in the U.S. parallels the introduction of triclosan, and because triclosan has two potential mechanisms by which it might alter human weight—*i.e.*, by modifying microbial flora and through endocrine disruption—we sought to assess the association between triclosan levels and body mass index (BMI) in three National Health and Nutrition Examination Surveys (NHANES) in which urinary triclosan and BMI, as well as relevant potential confounding variables, were simultaneously measured.

## Materials and Methods

The National Health and Nutrition Examination Survey (NHANES) is an ongoing federally-conducted “program of studies designed to assess the health and nutritional status of adults and children in the United States” [26]; all data are publically available. Approximately 5000 people selected to represent the U.S. population are studied each year using demographic, socioeconomic, anthropometric and biological measures. For this study, we combined the 2003–2004, 2005–2006, and 2007–2008 cohorts, all three of which measured triclosan levels in urine. All adults aged 20 years and older who provided information for four datasets (demography, anthropometry, environmental phenols, and cotinine) were included. We conducted sub-analyses on subsets of this population for which other information of interest was collected including: urinary bisphenol A (BPA); urinary 2,4-dichlorophenol; vitamins A and E, and carotenoids (available from 2003–2006 only); and specific gravity (available from 2007–2008 only). Pregnant subjects were excluded as was one outlier with BMI>100 kg/m^2^.

Survey methods have been described elsewhere [26]. In brief, demographic and anthropometric information were collected in a questionnaire and physical exam. Urinary triclosan levels were measured using solid phase extraction coupled to high-phase liquid chromatography and tandem mass spectrometry. BPA was measured using gas chromatography or high performance liquid chromatography coupled with different detection techniques [27]; measurement of 2,4-dichlorophenol was similar [28]. Creatinine was measured using commercially available analyzers [29] and cotinine was measured using isotope dilution-high performance liquid chromatography/atmospheric pressure chemical ionization tandem mass spectrometry [30].

We assessed effects of triclosan on BMI with adjustment for potential confounders including: survey year, sex, age, race, socioeconomic status (SES) as measured by the poverty index ratio (PIR). Because smoking is linked to weight [31], we also included cotinine level in the analysis. We computed Spearman's correlation between triclosan and 60 other urine or serum-based biomarkers of exposure that were measured in both the 2003–2004 and 2005–2006 surveys for subjects who also had triclosan measured [32]. In total, 132 markers of exposure overlapped between the 2003–2004 and 2005–2006 surveys. Of these 132, 60 were assayed on the same participants as triclosan (Table S1). Factors with greater than a 0.2 Spearman correlation coefficient were also tested in our model as possible confounders. Because BPA has been associated with higher BMI in NHANES [33], [34] with some correlation with triclosan (ρ = 0.1), we also elected to test it in some models.

Triclosan levels were nonlinear with approximately one-quarter of subjects having levels of triclosan below detectable; we therefore categorized triclosan in two ways: present vs. absent and by modified quartiles (those with a below-detection level of triclosan comprised the first “quartile”, and the remainder were divided into tertiles). Prior to division into quartiles, we adjusted for urinary creatinine using the ratio of triclosan to urinary creatinine. BPA and 2,4-dichlorophenol were categorized similarly to triclosan, also due to non-linearity (non-detectable and tertiles, with adjustment for creatinine). Cotinine was categorized as not detected, low (<12 ng/ml), and high (≥12 ng/ml); these cutoffs were previously shown to distinguish non-smokers, passive smokers and active smokers [35]. SES was categorized into three groups (PIR≤2, 2<PIR<5, and PIR≥5), based on the distribution of the data which were continuous up to poverty index ratio (PIR)<5 with all others categorized as PIR≥5. Race was categorized as Black non-Hispanic, White non-Hispanic, Hispanic, and Other/Multi-race.

Univariate distributions included medians and interquartile ranges for continuous variables and percentiles for categorical variables, as appropriate. Multivariate analyses were conducted using linear regression models of BMI on triclosan and the potential confounders as above. Triclosan was included in separate models as either a binary variable or in its ratio with urinary creatinine, as a categorical variable, due to the non-linearity of triclosan concentration distribution. Secondary analyses included urinary creatinine as a covariate and a sub-analysis excluding creatinine but using specific gravity as a covariate. Based on the correlations mentioned above, we also added BPA to the triclosan model and additionally ran models that substituted triclosan with its potential metabolite, 2,4-dichlorophenol [36], [37], [38], [39].

All biologically and demographically plausible interactions were considered in the final model. All models used the SAS survey-adjusted regression procedure (SAS 9.2, SAS Institute, Cary, NC), with estimates of beta and/or associated p values presented, as appropriate.

## Results

The three cohorts contributed 4701 eligible subjects; of these, 4037 with information on all variables in the primary model were included in the analyses (Table 1). Subjects were relatively uniformly distributed by age, ranging from 20 to 85 years. Approximately half of subjects were White non-Hispanic with other race groups oversampled relative to their U.S. population proportion. The median household income was 2.3 times that of poverty level. Median BMI was 27.8 kg/m^2^ for both sexes, with slightly larger variation in women.

**Table 1:** Descriptive statistics of NHANES subjects (N = 4037) included in analyses.

Parameter	N(%) or Median(IQR^a^)
**NHANES**	
2007–2008	1551 (38.4)
2005–2006	1221 (30.2)
2003–2004	1265 (31.3)
**Sex**	
Male	2058 (51.0)
Female	1979 (49.0)
**Race/ethnicity**	
Black non-Hispanic	810 (20.1)
Hispanic	979 (24.3)
White non-Hispanic	2083 (51.6)
Other/multi-race	165 (4.1)
**Socioeconomic status**	
High (PIR^b^≥5)	746 (18.5)
Medium (2<PIR<5)	1486 (36.8)
Low (PIR≤2)	1805 (44.7)
**Age (years)**	49 (35–65)
**Body mass index (kg/m^2^)**	27.78 (24.26–32.13)
**Cotinine (ng/mL)**	0.075 (0.020–33.6)
High (>12)	1090 (27.0)
Low (0.015–12)	2238 (55.4)
Below detection (<0.015)	709 (17.6)
**Triclosan (ng/mL)**	11.6 (2.70–61.4)
High (58.4–3620)	1046 (25.9)
Medium (10.6–58.3)	1034 (25.6)
Low (2.3–10.5)	1040 (25.8)
Below detection (<2.3)	917 (22.7)
**Triclosan (ng/mg creatinine)**	
High (52.029–3387)	1049 (26.0)
Medium (9.822–51.967)	1036 (25.7)
Low (.0699–9.821)	1035 (25.6)
**2,4-dichlorophenol (µg/L)**	1.0 (0.4–2.6)
High (2.2–933)	1162 (28.8)
Medium (0.8–2.1)	1157 (28.7)
Low (0.2–0.7)	1223 (30.3)
Below detection (<0.2)	495 (12.3)
**2,4-dichlorophenol (µg/mg creatinine)**	
High (1.742×10^−3^–0.088)	1179 (29.2)
Medium (6.635×10^−4^–1.739×10^−3^)	1192 (29.5)
Low (5.74×10^−5^–6.627×10^−4^)	1171 (29.0)

a) IQR: interquartile range.

b) PIR: poverty index ratio.

Triclosan was detectable in 77% of subjects, with a higher percentage in the most recent surveys (NHANES 2005–2006 (80%) and NHANES 2007–2008 (79%) compared to NHANES 2003–2004 (73%), *p*<0.001). Median triclosan level was 11.6 ng/mL.

In the multivariate linear regression model including detectable vs. non-detectable triclosan, a detectable level of triclosan was associated with an increase of 0.94 BMI points compared to below-detectable triclosan exposure, with a similar effect when BPA was included (*p*<0.001, Table 2). In the model with triclosan categorized into quartiles, the second and third quartiles of triclosan were associated with significant increases of 1.53 and 1.04 BMI points, respectively (*p*<0.001 and 0.002, Table 3). The highest quartile of triclosan did not have a significant increase in BMI (+0.26 BMI, *p* = 0.33). Again effects were similar with the addition of BPA to the model (Table 3).

**Table 2:** Multivariate linear regression results on outcome variable BMI (kg/m^2^) with triclosan and bisphenol A (BPA) categorized as detectable vs. non-detectable.

		*Model 1*		*Model 2*	
Parameter	Category	Estimate (95% CL)	*p*- value	Estimate (95% CL)	*p*- value
**Intercept**	-	26.20 (24.76, 27.65)	<0.001	26.26 (24.87, 27.65)	<0.001
**Triclosan/creatinine**	Detected	0.94 (0.48, 1.40)	<0.001	0.83 (0.40, 1.27)	<0.001
	Below detection	0	ref.	0	ref.
**NHANES**	2007–2008	0.79 (0.11, 1.46)	0.02	0.80 (0.15, 1.44)	0.02
	2005–2006	0.59 (−0.20, 1.38)	0.14	0.64 (−0.13, 1.41)	0.11
	2003–2004	0	ref.	0	ref.
**Cotinine**	High	−0.29 (−1.19, 0.60)	0.51	−0.38 (−1.24, 0.48)	0.39
	Low	1.23 (0.49, 1.96)	0.002	1.13 (0.43, 1.83)	0.003
	Below detection	0	ref.	0	ref.
**BPA/creatinine**	Detected	-	-	1.63 (0.91, 2.36)	<0.001
	Below detection	-	-	0	ref.
**Age (years)**	-	0.02 (0.009, 0.04)	0.001	0.02 (0.01, 0.04)	<0.001

Model 1, without BPA; Model 2,with BPA. Models (1/2) also included sex (*p* = 0.70/0.67), race (*p*<0.001/<0.001), socioeconomic status (SES) as measured by poverty index ratio (PIR) (*p* = 0.20/0.16), sex-race interaction (*p*<0.001/<0.001), and sex-SES interaction (*p* = 0.005/0.007).

**Table 3:** Multivariate linear regression results on outcome variable BMI (kg/m^2^) with triclosan and bisphenol A (BPA) categorized by quartile.

		*Model 1*		*Model 2*	
Parameter	Category	Estimate	*p*- value	Estimate	*p*- value
**Intercept**	-	25.29 (23.71, 26.87)	<0.001	25.92 (22.14, 25.69)	<0.001
**Triclosan/creatinine**	High	0.26 (−0.27, 0.80)	0.33	0.19 (−0.32, 0.71)	0.47
	Medium	1.04 (0.41, 1.68)	0.002	0.95 (0.32, 1.57)	<0.001
	Low	1.53 (0.85, 2.21)	<0.001	1.37 (0.74, 2.01)	<0.001
	Below detection	0	ref.	0	ref.
**NHANES**	2007–2008	0.76 (0.09, 1.44)	0.03	0.73 (0.10, 1.37)	0.03
	2005–2006	0.61 (−0.17, 1.39)	0.12	0.60 (−0.15, 1.34)	0.12
	2003–2004	0	ref.	0	ref.
**Cotinine**	High	−0.37 (−1.26, 0.51)	0.40	−0.44 (−1.28, 0.41)	0.32
	Low	1.18 (0.46, 1.91)	0.002	1.18 (0.46, 1.91)	0.003
	Below detection	0	ref.	0	ref.
**BPA/creatinine**	High	-	-	1.42 (0.59, 2.25)	0.002
	Medium	-	-	1.45 (0.71, 2.20)	<0.001
	Low	-	-	1.79 (0.99, 2.58)	<0.001
	Below detection	-	-	0	ref.
**Age (years)**	-	0.02 (0.009, 0.04)	0.002	0.02 (0.01, 0.04)	<0.001

Model 1, without BPA; Model 2,with BPA. Models (1/2) also included sex (*p* = 0.68/0.62), race (*p*<0.001/<0.001), socioeconomic status (SES) as measured by poverty index ratio (PIR) (*p* = 0.16/0.14), sex-race interaction (*p*<0.001/<0.001), and sex-SES interaction (*p* = 0.006/0.007).

**Figure 1:**
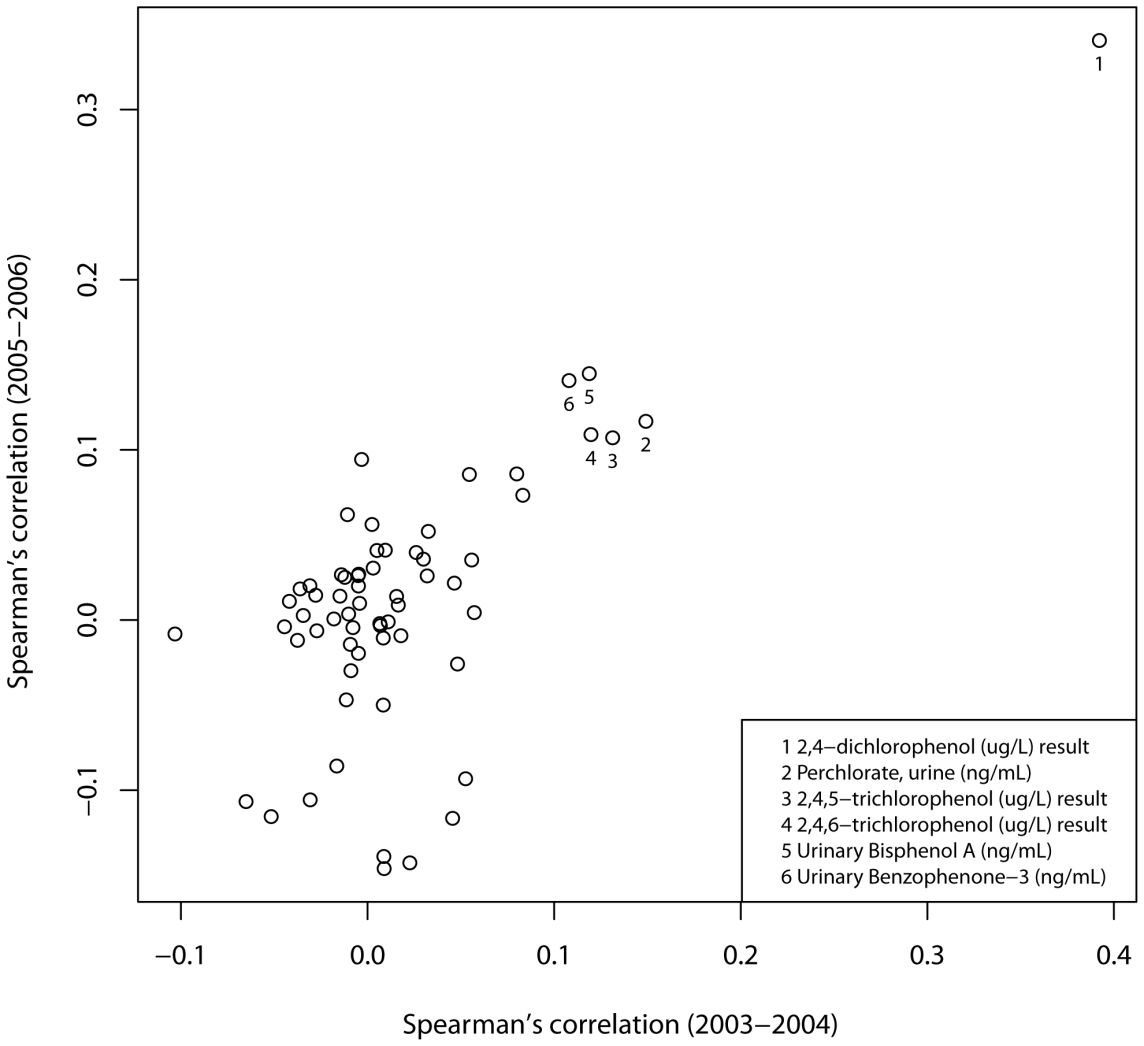
Correlations between triclosan and other biomarkers of exposure. Spearman correlations between urinary triclosan and 63 other biomarkers of exposure in the 2003–2004 (horizontal axis) and 2005–2006 (vertical axis) NHANES (National Health and Nutrition Examinations Surveys) survey cohorts. Correlations greater than 0.1 in both surveys are annotated (numbers 1–6).

The Spearman correlation coefficient between triclosan and one of its metabolites—2,4-dichlorophenol—was 0.4 (*p*<0.001) (Figure 1). We tested this metabolite using the identical model and found similar patterns to triclosan, with parameter estimates slightly smaller (binary model: +0.8 BMI, *p* = 0.003, quartiles model: +1.34, 0.6, 0.44 BMI, *p*<0.001, *p* = 0.04, 0.26 for the second, third, and highest quartiles, respectively). None of the other urine biomarkers tested correlated with triclosan with a coefficient as large. BPA and triclosan correlated at ρ = 0.1 (*p*<0.001) but inclusion of BPA in the model neither altered the magnitude nor the significance of triclosan's effects. As previously reported by others [33], [34], however, BPA was associated with a significant increase in BMI, in both the binary model (detection +1.6 BMI, *p*<0.001) and the quartile model (+1.8, 1.4, 1.4 BMI with *p* = 0.002, 0.017, 0.015 at the second, third, and highest quartiles, respectively).

BMI was significantly correlated with both urinary creatinine (ρ = 0.17, *p*<0.001) and specific gravity (ρ = 0.22, *p*<0.001). Without any correction for hydration (*i.e.*, neither creatinine nor specific gravity included in the models), results were quite similar to the adjusted findings (data not shown). Using creatinine as a covariate rather than in the triclosan/creatinine ratio also yielded similar results (data not shown). In the sub-analysis using specific gravity as a covariate, however, detectable triclosan was no longer significantly associated with BMI and point estimate were slightly attenuated, although the outcome of the effect was unchanged.

## Discussion

In this study, we identified a significant association between urinary triclosan and increased BMI after adjustment for known confounders. Those subjects with detectable triclosan in their urine had a large increase in BMI (+0.9 kg/m^2^); when analyzed by quartile, however, the dominant effect seemed to be at the second and third quartiles, rather than the highest one.

Studies on triclosan and body weight are sparse. In a small study in baboons (three exposed animals), no alteration in weight was observed at low or high doses of ingestion [40]. Triclosan can be detected in plasma and urine within hours of exposure. Median half-life has been found to be 26 minutes in saliva [41], 51 hours in plasma, and 65 hours in urine, so that within days it is cleared from the human body [42]. Although it is fat soluble, it is rapidly metabolized and there is no evidence of significant storage in body fat. In an analysis in an NHANES study of BPA in children, investigators found no significant association between urinary triclosan and obesity [34]. Interestingly, the supplemental tables do demonstrate a similar pattern to our observations with children in the second and third quartiles of triclosan somewhat more likely to be overweight or obese than those in the first and fourth quartiles; data were not presented, however, on BMI as a continuous outcome. Triclosan remains a plausible cause of weight gain. Triclosan is a biocide that kills a broad spectrum of microbial agents. In trials of toothpastes, triclosan is known to alter the oral flora [2]. Whether it also changes the flora of the gut remains unknown, although the fact that it is found in the blood indicates that, at the very least, it reaches the small bowel where it can be absorbed. Antibiotics, which are routinely integrated into feed, increase body mass in animals, and sparse data among military recruits suggest similar effects in humans [43]. Recent animal studies indicate that antibiotic use changes the gut flora which, in turn, alters gastrointestinal metabolism [24]. All told, triclosan, which is present in 75% of urine samples at a random point in time, is a much more constant exposure in humans than antibiotics. If antibiotics—which are rare exposures in most adults and infrequent even in children—are plausible as a cause of weight gain, then triclosan may be even more relevant through a similar mode of action.

In addition to interaction with the gut flora, triclosan or its metabolites have been postulated to act as endocrine disruptors. Among the endocrine effects, the one with most traction is interference with thyroid function; in vitro and in animals, studies have indicated both decreased synthesis and increased catabolism of thyroid hormone, resulting in lower levels of circulating thyroid hormone [12], [44], [45], [46]. The structure of triclosan resembles that of the non-steroidal estrogen, diethylstilbestrol (DES) [47], and a small number of animal studies have suggested it binds to both estrogen and androgen receptors, mimicking some of their activities and inhibiting others [11], [48]. In one study in Wistar rats, triclosan both potentiated the effects of estrogen on the age of onset of vaginal opening and on uterine weight, and also decreased thyroid hormone concentrations. Both decreases in thyroid hormone and increases in sex hormones can increase weight. However, the plasma levels of triclosan necessary to cause endocrine disruption in animals are well beyond those observed in humans [10]. Indeed, data on endocrine disruption are lacking, with the only published studies to date demonstrating no effect of triclosan on measures of thyroid function [49], [50]. Much more work needs to be done.

We found no clear dose response relationship between triclosan and BMI; rather, we observed the greatest effect at modest levels. It is possible that triclosan affects some microbial colonizers at low doses and others at high doses and that the different resulting microbial communities signal gut metabolism differently. Alternatively, triclosan has several mechanisms of action that interact with one another, *e.g.*, adding endocrine effects to microbial ones at higher doses. We also cannot exclude confounding of triclosan by dietary or environmental factors. Although we found no likely confounders within the NHANES dataset other than BPA, many other chemicals and environmental exposures remain unstudied.

One challenge of our analysis was determining a priori the best way to adjust for the concentration of the urine since analyte concentrations are higher in the setting of concentrated urine. To address this, some investigators have included urinary creatinine concentration as an independent variable in the model while others have used divided their urinary analyte by creatinine. We were concerned that creatinine, which is a product of muscle mass, would be associated with weight and therefore might be an inappropriate control in a study of BMI; indeed, a significant association between urinary creatinine and BMI was observed. Specific gravity, however, was even more strongly associated with body mass. In the end, these concerns were unwarranted since all methods tested—no adjustment, urinary creatinine as an independent variable, urinary triclosan divided by creatinine, and urinary specific gravity—yielded similar magnitudes of effect.

Not surprisingly, of the 60 urine biomarkers tested for correlation with triclosan, the one most highly correlated was 2,4-dichlorophenol, a triclosan metabolite. Like triclosan, 2,4-dichlorophenol is thought to have endocrine disruptive effects, particularly on the estrogen receptor [51]. Using this chemical as a surrogate for triclosan produced results with the same pattern, but slightly weaker than those with triclosan. The similarity of 2,4-dichlorophenol's relationship with BMI to that of triclosan ensures that we are likely measuring the effect of triclosan exposure and not that of triclosan waste or processing efficiency. Another triclosan metabolite that has been reported to have endocrine disruptive potential, 2,4,6-trichlorophenol, was more weakly correlated with triclosan and was not included in our model. Also weakly correlated with triclosan was BPA which, though associated with obesity, did not alter the observed effects of triclosan.

Obesity has increased dramatically over the last 50 years. Coincident with this rise have been dramatic changes in infectious diseases due to myriad new vaccines, decreases in family size, improved sanitation and hygiene and widespread antibiotic use. If common infectious diseases are less frequent, it is not unreasonable to assume that our colonizing flora have altered in parallel. In fact, we may well be seeing changes in the human micro-ecosystem that rival or exceed the degree of change in macro-ecosystems of rain forests and oceans. Recently, investigators have invoked the hygiene hypothesis—*i.e.*, that that hyper-cleanliness and absence of exposure to a broad range of microbial agents in early life suppress normal maturation of the immune response—as the link between allergic diseases and obesity [52], [53]. These data, together, suggest that the modern approach to household and personal cleanliness symbolized by household use of triclosan has may have unintended health consequences.

## Conclusions

Triclosan exposure is associated with increased BMI in adults in NHANES data. Stronger effects at moderate rather than high levels suggest a complex mechanism of action. Studies that elucidate triclosan's effect on microbial flora and on host and microbial metabolism can help determine mechanisms, if any, by which this chemical could be altering human growth and wellbeing.

## Supporting Information

Table S1 **Spearman correlation coefficients between triclosan and 60 variables for subjects with both measurements in survey years 2003–2004 and 2005–2006.**
(DOCX)
