# Supplementary material for: Urinary Triclosan is Associated with Elevated Body Mass Index in NHANES
Source: PLoS One. 2013 Nov 21;8(11):e80057. doi: 10.1371/journal.pone.0080057 (PMC3836985; doi:10.1371/journal.pone.0080057)
Supplement: Table S1 — Spearman correlation coefficients between triclosan and 60 variables for subjects with both measurements in survey years 2003–2004 and 2005–2006. (DOCX) [file pone.0080057.s001.docx]

**Online tables**

Table S1

| **Variable** | **Correlation (2003-2004)** | **Correlation (2005-2006)** |
| --- | --- | --- |
| Hepatitis B surface antigen | -0.028 | 0.015 |
| Hepatitis C antibody (confirmed) | -0.027 | -0.006 |
| Blood 2,5-Dimethylfuran (ng/mL) | 0.009 | -0.146 |
| a-Carotene(ug/dL) | 0.026 | 0.040 |
| Vitamin B12, serum (pg/mL) | -0.045 | -0.004 |
| Cadmium (ug/L) | -0.011 | -0.047 |
| trans-b-carotene(ug/dL) | -0.005 | 0.020 |
| Lead (ug/dL) | -0.052 | -0.115 |
| cis-b-carotene(ug/dL) | -0.014 | 0.027 |
| Cotinine (ng/mL) | -0.016 | -0.086 |
| b-cryptoxanthin(ug/dL) | 0.002 | 0.056 |
| Folate, serum (ng/mL) | -0.042 | 0.011 |
| g-tocopherol(ug/dL) | -0.009 | -0.030 |
| Hepatitis A Antibody (Anti-HAV) | -0.034 | 0.003 |
| Hepatitis B core antibody | -0.009 | -0.014 |
| Hepatitis B Surface Antibody | -0.005 | 0.026 |
| Herpes I | -0.005 | 0.027 |
| Herpes II | -0.038 | -0.012 |
| Mercury, inorganic (ug/L) | 0.011 | -0.001 |
| Iron, Frozen Serum (ug/dL) | 0.007 | -0.002 |
| Combined Lutein/zeaxanthin (ug/dL) | -0.010 | 0.003 |
| trans-lycopene(ug/dL) | 0.056 | 0.035 |
| Folate, RBC (ng/mL RBC) | -0.015 | 0.014 |
| Retinyl palmitate(ug/dL) | 0.003 | 0.031 |
| Retinyl stearate(ug/dL) | 0.018 | -0.009 |
| Mercury, total (ug/L) | 0.046 | 0.022 |
| Blood trans-1,2-Dichloroethene (ng/mL) | -0.103 | -0.008 |
| Blood Tetrachloroethene (ng/mL) | 0.010 | 0.041 |
| Blood Bromoform (pg/mL) | 0.048 | -0.026 |
| Blood Bromodichloromethane (pg/mL) | 0.030 | 0.036 |
| Blood Benzene (ng/mL) | 0.009 | -0.139 |
| Blood Chloroform (pg/mL) | 0.007 | -0.003 |
| Blood Dibromochloromethane (pg/mL) | 0.032 | 0.026 |
| Blood 1,4-Dichlorobenzene (ng/mL) | 0.016 | 0.009 |
| Blood Ethylbenzene (ng/mL) | 0.046 | -0.117 |
| Retinol(ug/dL) | 0.057 | 0.004 |
| Vitamin C (mg/dL) | -0.018 | 0.001 |
| a-Tocopherol(ug/dL) | 0.008 | -0.010 |
| Blood Methylene Chloride (ng/mL) | -0.004 | 0.010 |
| Blood MTBE (pg/mL) | -0.008 | -0.004 |
| Blood o-Xylene (ng/mL) | 0.023 | -0.143 |
| Blood Styrene (ng/mL) | -0.031 | -0.106 |
| Blood Trichloroethene (ng/mL) | 0.016 | 0.014 |
| Blood Toluene (ng/mL) | 0.008 | -0.050 |
| Blood m-/p-Xylene (ng/mL) | 0.053 | -0.093 |
| Water Bromoform (ng/mL) | -0.003 | 0.094 |
| Water Chloroform (ng/mL) | -0.012 | 0.025 |
| Water Dibromochloromethane (ng/mL) | -0.011 | 0.062 |
| Water MTBE (ng/mL) | 0.032 | 0.052 |
| 2,5-dichlorophenol (ug/L) result | 0.083 | 0.073 |
| 2,4,5-trichlorophenol (ug/L) result | 0.131 | 0.107 |
| 2,4,6-trichlorophenol (ug/L) result | 0.120 | 0.109 |
| Urinary 4-tert-octyl phenol (ng/mL) | 0.055 | 0.085 |
| Urinary Benzophenone-3 (ng/mL) | 0.108 | 0.141 |
| Urinary Bisphenol A (ng/mL) | 0.119 | 0.145 |
| 2,4-dichlorophenol (ug/L) result | 0.392 | 0.341 |
| O-Phenyl phenol (ug/L) result | 0.080 | 0.086 |
| Urinary Chlamydia | -0.065 | -0.107 |
| Urinary Gonorrhea | -0.036 | 0.018 |
| Perchlorate, urine (ng/mL) | 0.149 | 0.117 |
